# Supplementary material for: Neuron-specific enolase and neuroimaging for prognostication after cardiac arrest treated with targeted temperature management
Source: PLoS One. 2020 Oct 1;15(10):e0239979. doi: 10.1371/journal.pone.0239979 (PMC7529296; doi:10.1371/journal.pone.0239979)
Supplement: Attachment — Submitted filename: Response to Reviewers.docx [file pone.0239979.s002.docx]

**Reviewer #1: Thank you for giving me the opportunity to review your work. This is an interesting study investigating a clear knowledge gap in the field of post-resuscitation care.**

**1. There are several linguistic issues in the manuscript. It needs editing by a native English speaker. Although a reviewer is not supposed to provide secretarial support to the authors, I suggest that you type "neurological" instead of "neurologic" throughout your manuscript.**
🡪 Thank you for your comments. Originally submitted papers were revised in English by the American Journal Experts. However, according to your recommendation, I got an English proofreading once again from the American Journal Experts. And I switched to neurological instead of neurologic according to your recommendation.

**2. The title of your paper is confusing. Please keep it simple and catchy.**
🡪 I absolutely agree with your comments. I changed the title as follows.

Correlation between neuron-specific enolase and neuroimaging and their prognostic utility after cardiac arrest treated with targeted temperature management 🡪 Neuron-specific enolase and neuroimaging for prognostication after cardiac arrest treated with targeted temperature management

**3. In your abstract methods state that it is a retrospective study.**
🡪 Thank you for your comments. I changed the sentence as follows.

A retrospective observational cohort study was performed on patients who underwent at least one NSE measurement between 48 and 72 hr; received both a brain computed tomography (CT) scan within 24 hr and diffusion-weighted magnetic resonance imaging (DW-MRI) within 7 days after return of spontaneous circulation (ROSC); and were treated with TTM after out-of-hospital CA between 2009 and 2017 at the Seoul St. Mary’s Hospital in Korea.

**4. Your introduction is appropriate.**
🡪 Thank you for your comment.

**5. Methods: Please elaborate which sedatives and paralytic agents were used in the studied patients.**

🡪 Thank you for your comments. Midazolam and rocuronium were used for shivering control in our hospital. I added this in the Methods section.

Sedative (midazolam) and paralyzing (rocuronium) agents were immediately administered to control shivering before induction, followed by continuous infusion during the entire TTM phase.

**6. More details must be given about PV400 and ADC values. Not all readers are familiar with these imaging parameters.**
🡪 Thank you for your comments. I absolutely agree with your comments. I added the following sentences in the Methods section.

The ADC is a measure of the diffusion coefficient of water molecules within tissue and is calculated using MRI with DW imaging.

The % voxels of a low ADC value (PV), meaning the percentage of voxels below different ADC thresholds, was calculated.

**7. Results: Please include a figure - flowchart of your study.**
🡪 Thank you for your comments. I added a figure as you recommended.

**8. Discussion: Your discussion is weak. I suggest that you add a paragraph to briefly discuss other biomarkers for prognostication after cardiac arrest with emphasis to recent articles and in biomarkers for very early prognostication (before 48-72h). Is NSE better? Why it is better? Do we really need biomarkers?**
🡪 Thank you for your comments. I absolutely agree with your comments. Biomarkers have following advantages over other prognostic tools; objective, unaffected by sedation, ease of availability (bedside), ease of sampling and no expert skills needed. Therefore, there are still several limitations, but research on biomarkers must be continued and efforts to find an ideal biomarker must be continued. So far, NSE is the most used, but as you pointed out, a new biomarker study was recently published. I added a new paragraph as follows according to your comments.

Biomarkers have several advantages. They can be measured at the bedside, are not affected by sedatives and provide a numeric result with little space to interpret. Higher brain biomarker levels indicate more damage to neurons. However, they cannot indicate which part of the brain is most affected. Recently, novel biomarkers, such as serum tau and serum neurofilament light chain (NFL), have been studied, and some data have been published. In a substudy of a TTM trial, Mattsson et al. stated that higher serum levels of tau at 24, 48 and 72 hr after CA correlated with poor neurological outcomes after CA, and serum tau more accurately predicted a poor outcome than did serum NSE [38]. In another substudy of the TTM trial, Moseby-Knappe et al. stated that serum NFL levels at 24, 48 and 72 hr after CA were highly predictive markers of a long-term poor neurological outcome after CA [39]. Both studies used a single molecule array (SIMOA) assay, which is able to accurately quantify proteins or peptides present at low femtomolar concentrations, which are difficult to quantify using conventional techniques. However, SIMOA assays are not yet available for clinical use in most hospitals.

**9. I appreciate your list of limitations of your study.**
🡪 Thank you for your comments.

**10. Conclusion: I believe that you must also refer to the timing of NSE + MRI combination based on your findings.**

🡪 Thank you for your comments. I added the timing of parameters in Conclusion section as follows.

The GWR (≤ 24 hr) is weakly correlated with the mean ADC (≤ 7 days) and NSE (highest between 48 and 72 hr). The combination of a DWI parameter and NSE has better prognostic performance than the combination of the GWR and NSE. The addition of the GWR to a DWI parameter and NSE does not improve the prediction of neurological outcomes after CA treatment with TTM.

**Reviewer #2: Very nice manuscript. Very interesting topic.
Nevertheless there are some points that need revision/clarification
1. In the abstract (method section), please clarify the type of observational study (case-control) and also the place it took place (hospital) - both in page 1 and 8**

🡪 Thank you for your comments. I changed the abstract as following.

A retrospective observational cohort study was performed on patients who underwent at least one NSE measurement between 48 and 72 hr; received both a brain computed tomography (CT) scan within 24 hr and diffusion-weighted magnetic resonance imaging (DW-MRI) within 7 days after return of spontaneous circulation (ROSC); and were treated with TTM after out-of-hospital CA between 2009 and 2017 at the Seoul St. Mary’s Hospital in Korea.

**2. In introduction section (Page 9) line 3 you should write more about the different studies of TTM and use those references also (10.1056/NEJMoa1906661, doi:10.1001/jama.2013.282173, DOI: 10.1016/j.resuscitation.2015.07.018).**

🡪 Thank you for your comments. I changed the sentence as follows and added the references as you recommended.

Targeted temperature management (TTM) between 32 and 36°C for at least 24 hr, for both shockable and nonshockable rhythms, is generally considered standard care after CA. Even though TTM can improve neurological outcomes and survival [2-8], two-thirds of patients will die after CA due to hypoxic-ischemic brain injury [9].

6. Lascarrou JB, Merdji H, Le Gouge A, et al. Targeted Temperature Management for Cardiac Arrest with Nonshockable Rhythm. N Engl J Med. 2019;381(24):2327-2337.

7. Kim F, Nichol G, Maynard C, et al. Effect of Prehospital Induction of Mild Hypothermia on Survival and Neurological Status Among Adults With Cardiac Arrest: A Randomized Clinical Trial. JAMA. 2014;311(1):45–52.

8. Nolan JP, Soar J, Cariou A, et al. European Resuscitation Council and European Society of Intensive Care Medicine Guidelines for Post-resuscitation Care 2015: Section 5 of the European Resuscitation Council Guidelines for Resuscitation 2015. Resuscitation. 2015;95:202-222.

**3. In introduction section (Page 9) lines 4-6, be careful... The main causes of death is not pour neurological outcome but withdrawal of care due to poor neurological outcome. Re-write the sentence and use more references (eg** [**https://doi.org/10.1016/j.resuscitation.2019.01.031**](https://doi.org/10.1016/j.resuscitation.2019.01.031)**)**

🡪 I absolutely agree with your comments. I changed the sentences as follow

However, the leading cause of death is the withdrawal of life-sustaining treatment (WLST) because of failure to awaken [10-12]. Therefore, special precautions should be taken to avoid false outcome predictions, which affect the decisions regarding WLST [9].

11. Witten L, Gardner R, Holmberg MJ, et al. Reasons for death in patients successfully resuscitated from out-of-hospital and in-hospital cardiac arrest. Resuscitation 2019;136:93-9.

**4. Please change “neurologic” to “neurological”.**

🡪 Thank you for your comment. I changed neurologic to neurological according to your recommendation.

**5. Page 9 Lines: "However, no single test can predict neurologic outcomes with a 0% false positive rate (FPR)", please determine if it is good, bad or overall neurological outcome.**

🡪 Thank you for your comment. I absolutely agree with your comment. There was some confusion. I wanted to say that in a patient treated with TTM, the prognosis cannot be determined by a single test. So, I changed the sentence as follow.

However, no single test can predict poor neurological outcomes with a 0% false-positive rate (FPR) in patients treated with TTM.

**6. Page 9 Last sentence ("we hypothesized..."): Lacks rationale behind the hypothesis. How did you get the main idea for that combination and why NSE and neuroimaging and not others? Is it due to center availability or other knowledge?**

🡪 Thank you for your comments. I absolutely agree with your comments. We previously reported that GWR can predict poor neurologic outcome after CA treated with TTM. And, we also reported that DW-MRI can predict poor neurologic outcome after CA treated with TTM. The NSE and S100 were available biomarker in our hospital. However, S100 was not recommended at the AHA and ERC guidelines, so we chose NSE as a biomarker to test. The purpose of this study was to determine whether combinations of multiple tests performed better than individual tests alone in patients treated with TTM after cardiac arrest. This study is a retrospective study. And the combination of NSE and neuroimaging showed the largest number of patients, which was an ideal model for testing this question. Therefore, it is because of center availability not for any special knowledge. I already mentioned in the limitation section that “Finally, we did not test other prognostic modalities, such as neurological examinations, SSEP, and EEG. Future studies should be conducted to determine the prognostic value of these modalities” So I added the following sentences.

We have previously reported that brain CT and brain DW-MRI can predict poor neurological outcomes in patients treated with TTM after CA [21, 29]. The purpose of this study was to determine whether combinations of multiple tests would perform better than individual tests in patients treated with TTM after CA. The combination of NSE and neuroimaging, which was used in the largest number of patients in our hospital, was an ideal model for testing this hypothesis.

**7. Page 10 Methods. Why those two time points (48 and 72hrs)? Why not only 72 hrs? Explain in methods.**

🡪 Thank you for your comments. The AHA guideline recommended that NSE and S100B should not be used alone to predict a poor neurologic outcome. It may reasonable to consider high serum values of NSE at 48 to 72 hrs after CA to support the prognosis of a poor neurologic outcome (Class IIb). Moreover, high NSE values between 48 and 72 hrs after ROSC were recommended for the prognostication strategy algorithm in the ERC guideline. And, The AUCs of NSE at 24, 48, 72 hrs in the TTM Trial were 0.75, 0.85 and 0.86, respectively. So, we thought that NSE values at 48 and 72 hr after ROSC have similar predictive values for predicting poor neurological outcomes. In the vast majority of patients, at least one sample was collected between 48 and 72 hrs after CA. So, we want to minimize the effect of missing data from a single time point. I added the sentence as follows.

In the majority of the patients, at least one sample was collected between 48 and 72 hr after ROSC, which minimized the effect of missing data from a single time point.

**8. Page 11 CT section: average GWR is not the best measurement to choose. The best seems to be Basal Ganglia GWR and not aGWR (10.1016/j.resuscitation.2018.08.024). I strongly recommend to leave CT measurements out of the study cause they are misleading and may extrapolate wrong conclusions about the use of CT in CA.**

🡪 Thank you for your comments. I absolutely agree with your comments. Authors were serious about your opinion. Basal ganglia seems to be the best region to calculate GWR for predicting neurologic outcome after CA. The AUC of basal ganglia GWR for predicting neurologic outcome of our cohort is higher than that of cerebrum GWR and average GWR (0.625 vs 0.599 and 0.619). However, it was not statistically significant (basal ganglia vs average GWR, p=0.8286). Therefore, using the basal ganglia GWR instead of the average GWR does not change the results of this study. Moreover, the average GWR was the most used in the meta-analysis study you mentioned (average GWR 9 study, basal ganglia 7 study, cerebrum GWR 6 study). The purpose of this study is how the prognostic value changes when the brain CT (GWR) performed at an early stage is combined with other prognostic modalities.

As we mentioned in the Discussion section, brain CT is one of the most commonly used tools in real clinical practice for detecting intracranial abnormality and predicting neurologic outcome. Brain CT should be performed before TTM to detect intracranial abnormality. However, brain CT performed at an early stage (especially within 2 hrs) has a disadvantage of having low sensitivity. When brain CT is repeated, the predictive value increases [REF]. Therefore, when discussing the predictive power of brain CT for outcome prediction after cardiac arrest, the scan time is very important issue. In our study, brain CT was mostly performed within 2 hrs after ROSC. So, the predictive value was relatively low than other prognostic tools (AUC of aGWR 0.619, AUCs of Mean ADC, PV400and NSE 0.830, 0.882 and 0.912). In addition, the authors stated that “the addition of the GWR to a DWI parameter and NSE does not improve the prediction of neurological outcomes after CA treatment with TTM” in the Conclusion section. We didn’t mean that brain CT should not be used because it does not help with outcome prediction. This should be limited to brain CT performed early (within 2 hrs).

It is difficult to exclude Brain CT from our study according to your opinion. Instead, the following was added to the Discussion and limitation section to prevent readers from misleading the predictive value of brain CT as a result of this study.

[REF] Hong JY, Lee DH, Oh JH, et al. Grey-white matter ratio measured using early unenhanced brain computed tomography shows no correlation with neurological outcomes in patients undergoing targeted temperature management after cardiac arrest. Resuscitation. 2019;140:161-169. doi:10.1016/j.resuscitation.2019.03.039

In the Discussion section

Brain CT should be performed before TTM to detect intracranial abnormalities. However, brain CT performed at an early stage (especially within 2 hr) has the disadvantage of a low sensitivity [20-22]. When brain CT is repeated, the predictive value increases [32]. Therefore, when discussing the predictive power of brain CT for outcome prediction after CA, the scan time is a very important issue. In our study, brain CT was mostly performed within 2 hr after ROSC. Therefore, the predictive value was relatively low compared with that of other prognostic tools (AUC of a GWR of 0.619; AUCs of the mean ADC, PV400 and NSE of 0.830, 0.882 and 0.912, respectively). Furthermore, the addition of the GWR to a DWI parameter and NSE did not improve the prediction of neurological outcomes after CA treatment with TTM. We do not suggest that brain CT should not be used because it does not help with outcome predictions. This should be limited to brain CT performed early (within 2 hr).

In the limitation section

Sixth, basal ganglia seem to be the best region to calculate the GWR for predicting a neurological outcome after CA [42]. The AUC of the basal ganglia GWR for predicting the neurological outcomes of our cohort was higher than that of the cerebrum GWR and average GWR (0.625 vs. 0.599 and 0.619, respectively). However, the difference was not statistically significant (basal ganglia GWR vs. average GWR, p=0.8286). Therefore, using the basal ganglia GWR instead of the average GWR would not change the results of this study.

**9. Page 14 Results section: Please define the initial rhythm of the rest 66.1% (PEA vs Asystole). What was the cause of arrest if not cardiac?**

🡪 Thank you for your comments. I added the following in the Result section.

Ventricular fibrillation was the initial rhythm for 37 (33.9%) subjects. A pulseless electrical activity was observed in 23 (21.1%) subjects; an asystole was recorded in 45 (41.3%) subjects; and the initial rhythm was unknown in the remaining 4 (3.7%) subjects. The cardiac cause of arrest was diagnosed in 63 (57.8%) subjects. The rest had a respiratory cause of arrest (34 subjects, 31.2%), other medical causes (10 subjects, 9.2%), submersion (1 subject, 0.9%), drug intoxication (1 subject), and an unknown cause (1 subject).

**10. Page 18 Discussion session (LIMITATIONS): Please elaborate on NSE limitations (ie "Biomarker thresholds vary with timing of measurement, reflecting their kinetics following initial release. An additional cause of inconsistency is the variability of techniques used to measure biomarkers, which can cause a significant systematic error between techniques"DOI https://doi.org/10.1186/s13054-018-2060-7, DOI: 10.1186/1756-0500-7-726). Discuss about other biomarkers (that may be more accurate), like tau-proteins from blood sampling.**
🡪 Thank you for your comments. I absolutely agree with your comments. I added the following sentences in the Limitation section as you recommended.

Fifth, NSE has the following disadvantages. The NSE threshold is dependent on the timing of the measurement, reflecting the kinetics after the initial release. An additional cause of discrepancies is the variability of the technology used to measure biomarkers, which can lead to significant systematic errors between technologies [40, 41].

[40] Sandroni, C., D’Arrigo, S. & Nolan, J.P. Prognostication after cardiac arrest. Crit Care 2018;22:150.

[41] Rundgren, M., Cronberg, T., Friberg, H. et al. Serum neuron specific enolase – impact of storage and measuring method. BMC Res Notes 2014;7:726.

The overall idea and methods (besides CT measurements) are nice.

**Reviewer #3: I really enjoyed this article. Despite the retrospective design, it provides important data that will stimulate new RCTs. I agree with the authors that the ERC/ESICM prognostication algorithm needs further (and in-detail) testing.**

**1. Please add as a limitation that you only studied the target of 33 ºC and thus your findings cannot extrapolate to other TTM temperature targets.**

 🡪 Thank you for your comments. I absolutely agree with your comments. I added the following in the Limitation section.

 Third, only patients treated at 33°C were included in this study. Therefore, our findings cannot be extrapolated to patients who were treated with other TTM temperature targets.
